# The complete mtDNA genome of suspected natural Bagridae hybridization (*Pelteobagrus olimcorporis* sp.nov): genome characterization and phylogenetic analysis

**DOI:** 10.1080/23802359.2016.1214544

**Published:** 2016-09-05

**Authors:** Sha Li, Xueqing Liu, Wei Jiang, Yunqin Bai, Tao Huang, Junhong Wang

**Affiliations:** a Institute of Chinese Sturgeon, China Three Gorges Corporation, Yichang, China;; b Hubei Key Laboratory of Three Gorges Project for Conservation of Fishes, Institute of Chinese Sturgeon, China Three Gorges Corporation, Yichang, China

**Keywords:** Bagridae, suspected new species, complete mitochondrial, hybrid

## Abstract

The complete mitochondrial genome sequence of the suspected new species (*Pelteobagrus olimcorporis* sp.nov) was first determined by a PCR-based sequencing method in this study. The complete mtDNA genome of this suspected new species is 16,533 bp in length and consists of 13 protein-coding genes, 22 transfer RNA genes (tRNA), two ribosomal RNA genes (rRNA) and a non-coding control region (D-loop). Compared with the 13 proteins genome of the Bagridae and Cyprinidae, results showed that this suspected new species was clustered together and from a sister group with fishes of Bagridae. Blasting the special region on NCBI, we found 99% of the sequence was the same with *Pseudolaubuca sinensis*. It indicated that *P. olimcorporis* sp.nov is a hybrid and the mitochondrial genomes of the father may permeate to the hybrid.

Since the establishment of the Three Gorges project changed the water ecological environment of fish in the past, the number and structure of fish composition changed greatly. In order to understand the concrete impact degree to fishes in the Yangtze River by the project and take effective measures to protect these species, the Chinese sturgeon Research Institute carried out related research and investigation every year, such as the natural reproduction monitoring of Chinese sturgeon, releasing and track, ecological operation of Asian carp and catches investigation (Zhang et al. 2011).

During fishes investigation at Yichang MiaoZui pier (N 30°42′18.53″, E111°16′08.88″) in November 2015, we found one odd-looking specie and it was stored in 95% ethanol. This specimen was stored in Institute of Chinese Sturgeon with accession number: 20151121PO1. Morphological identification showed it may be one new species (*Pelteobagrus olimcorporis* sp.nov) that natural hybrid by Different kinds of Bagridae fishes. Total genomic DNA was extracted from caudal fins by a traditional phenol–chloroform method (Sambrook & Russell 2002). Its mitochondrial DNA was sequenced, analyzed and the complete mitogenome sequence has been deposited in GenBank with the accession number: KX347977. The results showed that the complete mtDNA genome of this suspected new species is 16,533 bp in length and consist of 13 protein-coding genes, 22 transfer RNA genes (tRNA), two ribosomal RNA genes (rRNA) and a non-coding control region (D-loop). The gene arrangement of this suspected new species is similar to other Bagridae (Dodson & Lecomte 2015).

To probe the phylogenetic relationship of this suspected new species, we selected six from the Bagridae family to reconstruct the phylogenetic tree with the neighbour-joining (NJ) method using the MEGA 6.0 (Kumar et al. 2008). The mtDNA sequences of 10 species of fishes were downloaded from GenBank, *Acipenser sinensis* (KJ174513.1) was used as an out-group for phylogenetic analysis. The tree topologies based on the 13 proteins in this study were identical and were statistically supported by high bootstrap and posterior probability values (Figure 1). The mitogenome data provided strong supported that this suspected new species was clustered together and from a sister group with *Leiocassis longirostris* (GU596454.1), *Pseudobagrus albomarginatus* (NC022726.1, *L. crassilabris* (JX867257.1), *P. nitidus* (HM746659.1) and *Pseudobagrus brevicaudatus* (NC021393.1), which belongs to Bagridae.

**Figure 1.**
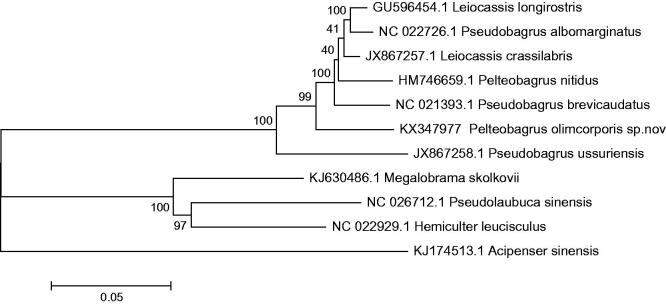
The consensus phylogenetic relationship of suspected natural Bagridae hybridization (*Pelteobagrus olimcorporis* sp.nov) with other Bagridae species. The numbers along the branches are Bayesian posterior probability and bootstrap values for NJ, estimated for concatenated mitochondrial protein sequences.

The result of sequence alignment on NCBI showed that the sequence similarity of this suspected new species and *L. crassilabris* can reach 97%. Then analyzing these two sequences by DANMAN 6.0, we found the sequence is obviously different from 10,000bp to 12,000bp which code ND4 and ND4L. Blasting this special region on NCBI, we found 99% of the sequence was the same with *Pseudolaubuca sinensis* (Chen et al. 2015). It indicated that *P. olimcorporis* sp.nov is a hybrid and the mitochondrial genomes of father may permeate to the hybrid. But it is still need further analysis to determine whether it is hybridize by *L. crassilabris* and *P. sinensis*. We expect that the present result can contribute to construct molecular identification of this species and be helpful to explore the phylogeny of Bagridae.
